# LncRNA-H19 Drives Cardiomyocyte Senescence by Targeting miR-19a/socs1/p53 Axis

**DOI:** 10.3389/fphar.2021.631835

**Published:** 2021-02-16

**Authors:** Yuting Zhuang, Tingting Li, Hongwen Xiao, Jiaxu Wu, Shuang Su, Xue Dong, Xiaoxi Hu, Qi Hua, Junwu Liu, Wendi Shang, Jiaming Ju, Fei Sun, Zhenwei Pan, Yanjie Lu, Mingyu Zhang

**Affiliations:** ^1^ Department of Pharmacology (State-Province Key Laboratories of Biomedicine-Pharmaceutics of China, Key Laboratory of Cardiovascular Medicine Research, Ministry of Education), College of Pharmacy, Harbin Medical University, Harbin, China; ^2^ China Northern Translational Medicine Research and Cooperation Center, Heilongjiang Academy of Medical Sciences, Harbin Medical University, Harbin, China

**Keywords:** p53, SOCS1, miR-19, cardiomyocyte senescence, H19

## Abstract

**Purpose: **Cardiomyocyte senescence is associated with a progressive decline in cardiac physiological function and the risk of cardiovascular events. lncRNA H19 (H19), a well-known long noncoding RNA (lncRNA), is involved in the pathophysiological process of multiple cardiovascular disease such as heart failure, cardiac ischemia and fibrosis. However, the role of H19 in cardiomyocyte senescence remains to be further explored.

**Methods: **Senescence-associated β-galactosidases (SA-β-gal) staining was used to detect cardiomyocyte senescence. Western blot, qRT-PCR and luciferase reporter assay were employed to evaluate the role of H19 in cardiomyocyte senescence and its underling molecular mechanism.

**Results: **H19 level was significantly increased in high glucose-induced senescence cardiomyocytes and aged mouse hearts. Overexpression of H19 enhanced the number of SA-β-gal-positive cells, and the expression of senescence-related proteins p53 and p21, whereas H19 knockdown exerted the opposite effects. Mechanistically, H19 was demonstrated as a competing endogenous RNA (ceRNA) for microRNA-19a (miR-19a): H19 overexpression downregulated miR-19a level, while H19 knockdown upregulated miR-19a. The expression of SOSC1 was dramatically increased in senescence cardiomyocytes and aged mouse hearts. Further experiments identified SOCS1 as a downstream target of miR-19a. H19 upregulated SOCS1 expression and activated the p53/p21 pathway by targeting miR-19a, thus promoting the cardiomyocytes senescence.

**Conclusion: **Our results show that H19 is a pro-senescence lncRNA in cardiomyocytes acting as a ceRNA to target the miR-19a/SOCS1/p53/p21 pathway. Our research reveals a molecular mechanism of cardiomyocyte senescence regulation and provides a novel target of the therapy for senescence-associated cardiac diseases.

## Introduction

Cardiomyocyte senescence is associated with abnormalities of cardiac performance and structure with declination of cardiac relaxation and contraction, increase in left ventricle weight and cardiomyocyte size, as well as excess interstitial extracellular matrix deposition. Cardiomyocyte senescence is not only a physiological process during the natural aging heart but also creates a lower threshold for many cardiac diseases to occur such as heart failure (Shah et al., 2015). At present, it is considered that oxygen free radical damage, telomere shortening and gene mutation are the main causes of senescence (Yuan et al., 2019; Oliveira de Almeida et al., 2020). Additionally, p53-p21 and p16^INK4A^-Rb have been recognized as the main molecular pathways for cell senescence (Rufini et al., 2013). Senescence is a complicated process involving a number of factors/pathways. Thus, deciphering the molecular mechanisms of cardiomyocyte senescence will be helpful to improve our understanding of aging-associated cardiac diseases and to find novel approaches for treatment.

As one of the intensively studied lncRNAs, H19 is a highly conversed lncRNA with a length of 2,288 nt. Emerging evidence demonstrates that H19 plays important roles in human cardiovascular diseases. It has been reported that H19 is involved in regulation of fetal and early postnatal growth control in human and other species (Gabory et al., 2010). Studies showed that H19 had significant diagnostic value for acute myocardial infarction (Wang et al., 2019b) and considered an independent predictor for cardiac artery disease (Zhang et al., 2017c; Xiong et al., 2019). H19 is a critical regulator of cardiomyocyte apoptosis and proliferation (Wang et al., 2019a; Li et al., 2019). H19 knockdown in dilated cardiomyopathy rats attenuates cardiomyocyte apoptosis and improves left ventricular structure and function (Zhang et al., 2017b). Knockdown of H19 in P19CL6 cells promotes cell proliferation and inhibits cell apoptosis during late-stage cardiac differentiation (Han et al., 2016). Silencing H19 was able to block the protecting actions of melatonin on senescent C-kit^+^ cardiac progenitor cells (Cai et al., 2016). Recent study showed that the expression of H19 is decreased during aging and H19 depletion in endothelial cells results in premature senescence via stimulation of STAT3 signaling (Hofmann et al., 2019). These studies demonstrated that H19 plays different role in different type of cells and different biological processes. However, whether H19 is involved in regulation of cardiomyocyte senescence and its underlying mechanisms remain unclear.

It has been shown that altered miRNA expression profiles are found in aging brain, lung and heart (Li et al., 2011; Verjans et al., 2017). Previous studies showed that the decreased expression of miR-19a contributed to increases in CTGF and TSP-1 expression in aging cardiomyocytes and failure-prone aging hearts in mice. Moreover, SOCS1 expression is also inhibited by miR-19a in human multiple myeloma (Pichiorri et al., 2008). MiR-19b expression is also significantly decreased in human age cells (Hackl et al., 2010). MiRNAs have been demonstrated as important regulators of cellular senescence and aging (Smith-Vikos and Slack, 2012; Dietrich et al., 2018). Accumulating evidence has revealed that miRNAs can be regulated by lncRNAs through sponging mechanism. MIAT regulates TGF-β1 expression by targeting miR-24 through complementary binding (Qu et al., 2017). LncRNA PCFL (pro-cardiac fibrotic lncRNA) contributes to myocardial infarction-induced cardiac fibrosis by acting as a ceRNA of miR-378 (Sun et al., 2019).

Senescent cells are characterized by increased senescence-associated β-galactosidase (SA-β-gal) activity and senescence proteins markers p53 and p21 (Collado et al., 2007). p53/p21 signaling pathway plays a central role in prompting cell senescence. Enhancing p53 activity could lead to senescence in cardiac fibroblasts, whereas inhibition of the p53 activity exhibits the opposite effect (Zhu et al., 2013). In addition, suppressor of cytokine signaling 1 (SOCS1) can act as a mediator for p53 phosphorylation and activation that is linked to oncogene-induced senescence (Ferbeyre et al., 2002).

The present work was designed to explore the role and potential mechanisms by which H19 regulates cardiomyocyte senescence. Our data show that H19 acts as competing endogenous RNA (ceRNA) of miR-19a to promote SOCS1 expression in cardiac senescence by stimulation of p53 and p21.

## Materials and Methods

### Isolation and Culture of Neonatal Mouse Ventricular Cells

All animal care and experimental procedures were approved by the Ethical Committee of Harbin Medical University for the Animal Care and Use. In the current study, the neonatal mice were purchased from the Animal Center of the Second Affiliated Hospital of Harbin Medical University. Neonatal mouse ventricular cells (NMVCs) were isolated from 1- to 3-day-old Kunming mice. The mice were sterilized with 75% ethanol before decapitated. The hearts were obtained and rapidly removed into DMEM (Dulbecco’s Modified Eagle Medium; Hyclone Laboratories, Utah, United Statees). The isolated hearts were cut into l∼3 mm pieces. The tissues were dissociated in 0.25% trypsin at 37°C and cell suspension was collected. After centrifugation (1,500 rpm, 5 min), the isolated cells were resuspended in DMEM containing 10% fetal bovine serum (Biological Industries, Kibbutz Beit Haemek, Israel), 100U/ml penicillin and 100 μg/ml streptomycin. Then, the cells were cultured in DMEM at 37°C in humidified air with 5% CO_2_ and 95% air for 1.5 h. After fibroblasts adherence, the non-adherent cardiomyocytes were uniformly replanted into a 6-well plate at a density of 1 × 10^6^ cells per well, and incubated in high glucose (25 mM) DMEM at 37°C in humidified air with 5% CO_2_ and 95% air.

### Transfection of H19 siRNAs

H19 siRNA (sense sequence: 5′-AUG​GGA​AUG​GUG​UGU​CUG​CTT-3′, antisense sequence: 5′-GCA​GAC​ACA​CCA​UUC​CCA​UTT-3′) and the negative control for H19 siRNA (sense sequence: 5′-UUC​UCC​GAA​CGU​GUC​ACG​UTT-3′, antisense sequence: 5′-ACG​UGA​CAC​GUU​CGG​AGA​ATT-3′) were designed and synthesized by Invitrogen (Shanghai, China). H19 siRNA or its negative control (100 nM) was mixed with Opti MEM^®^ I Reduced Serum Medium (Gibco, New York, United States) for 5 min and then with X-treme GENE siRNA Transfection Reagent (Roche, Mannheim, Germany) at room temperature for 20 min. The NMVCs were transfected with the mixture according to the manufacturer’s instruction followed by various designated treatments.

### Lentiviral Constructions and Infection

The full-length H19 cDNA was synthesized and subcloned into a third-generation replication-defective HIV-1-based lentiviral vector. This vector was packaged into viral particles in 293T cells by co-transfection with packaging-defective helper plasmids. Empty vector was packaged in 293T cells as vector control. Virus-containing supernatants were collected 48 h later, and the lentiviral titers were determined by high-content screening. When NMVCs were ready for transfection, 100 MOI lenti-H19 was added in medium. Non-transfected cells were regarded as a control. After transfection for 48 or 72 h, cells were collected for qRT-PCR and Western blot analysis.

### Transfection of miRNA Mimic and Its Inhibitor

miR-19a mimic (sequence: 5′-UGU​GCA​AAU​CUA​UGC​AAA​ACU​GA-3′) and its inhibitor AMO-miR-19a (sequence: 5′-UCA​GUU​UUG​CAU​AGA​UUG​CAC​A-3′) were synthesized by RiboBio (Guangzhou, China). Additionally, a scrambled sequence was used as a negative control (NC) (sequence: 5′-UUCUCCGAACGUGU CACGU-3) that was designed according to the BLAST search of the human/mouse genome. The X-treme GENE siRNA Transfection Reagent (Roche) was used for transfection. After transfection with miR-19a mimic (100 nm) or AMO-miR-19a (100 nm) for 72 h, the NMVCs were used for qRT-PCR and Western blot analysis.

### Western Blot Analysis

Total protein was extracted and mixed with 5 × loading buffer (Beyotime, Shanghai, China) at 100°C for 5 min. The concentration of cell lysis was determined by BCA kit (Beyotime) according to the instruction. For Western blot analysis, denatured protein samples (80–100 μg each) were subjected to 12% SDS-polyacrylamide gels and transferred to nitrocellulose membranes. Blots were probed using primary antibodies. Membranes were detected on Odyssey infrared scanning system (LI-COR Biosciences, Lincoln, United States), and the Western blot bands were quantified using Odyssey 3.0 software. The antibody against p21 was obtained from BD Pharmingen (BD Pharmingen, Franklin lakes, NJ). The antibody against p53 and phosphorylated p53 were obtained from Cell Signaling Technology (Cell Signaling Technology, Danvers, MA). The antibody against GAPDH was obtained from Kangcheng (Kangcheng Inc., Shanghai, China).

### Quantitative Reverse Transcription-PCR (qRT-PCR)

Total RNA was extracted from cultured cells using a Trizol standard protocol (Invitrogen, Carlsbad, United States). The quantity and purity of RNA were examined using Nano-Drop 8,000 Spectrophotometer (Thermo Scientific, Wilmington, United Staates). For each sample, 500 ng total RNA was reverse transcribed into cDNA with High Capacity cDNA Reverse Transcription Kit (TOYOBO, Osaka, Japan), and then amplified with SYBR Green I (TOYOBO, Osaka, Japan) using 7,500 Real Time-PCR System (Applied Biosystems, Foster City, CA, United States). The threshold cycle (Ct) was determined. Relative lncRNA and miRNA levels were calculated based on the Ct values and normalized to GAPDH or U6 level for each sample. The sequences of primers are shown in Table 1.

**TABLE 1:** The sequences of primers.

Gene name	primer	Sequence
H19	Forward	5’-TCA​TCA​TCT​CCC​TCC​TGT​CT-3’
	Reverse	5’-GGT​AAA​TGG​GGA​AAC​AGA​GT-3’
GAPDH	Forward	5’-AAG​AAG​GTG​GTG​AAG​CAG​GC-3’
	Reverse	5’-TCC​ACC​ACC​CAG​TTG​CTG​TA-3’
miR-19a	Forward	5’-GTG​GTG​TGC​AAA​TCT​ATG​CAA-3’
	Reverse	5’-CAG​TGC​GTG​TCG​TGG​AGT-3’
U6	Forward	5'-GCT​TCG​GCA​GCA​CAT​ATA​CTA​AAA​T-3'
	Reverse	5'-CGC​TTC​ACG​AAT​TTG​CGT​GTC​AT-3'

### Luciferase Reporter Assay

A fragment of the mouse H19 gene containing the miR-19a binding site (designated Luc-wt-H19) and another fragment with nucleotide replacement mutation (designated Luc-mut-H19) were PCR amplified. For luciferase reporter gene assay, HEK293 cells were cultured in 24-well culture plates and transfected with varying constructs. Firefly and Renilla luciferase activities were measured using the Dual Luciferase Reporter Assay System (Promega, Madison, WI, United States).

3′UTR of the mouse SOCS1 gene containing the miR-19a binding site (designated Luc-wt-SOCS1) and another fragment with nucleotide replacement mutation (designated Luc-mut-SOCS1) were PCR amplified. For luciferase reporter gene assay, HEK293 cells were cultured in 24-well culture plates and transfected with varying constructs. Firefly and Renilla luciferase activities were measured using the Dual Luciferase Reporter Assay System (Promega, Madison, WI, United States).

### Measurement of Senescence-Associated β-galactosidase Activity

Activity of senescence-associated β-galactosidase (SA-β-gal) in NMVCs was analyzed using senescence β-galactosidase staining kit (Genmed Scientifics Inc., Boston, United States) according to the manufacturer’s instructions. To quantify the percentage of SA-β-gal-positive cells, digital images of 10 randomly chosen fields were captured by a microscope and the cells from each sample were counted for determining the percentage of senescent cells.

### Statistical Analysis

All data are presented as mean ± SEM. The Student’s t-test was used for comparisons between two groups. The one-way or two-way analysis of variance (ANOVA) was adopted for comparisons of multiple groups. A value with *p* < 0.05 was considered statistically significant.

## Results

### Senescence Promoted H19 Expression in Both Cultured NMVCs and Aged Mouse Hearts

**FIGURE 1:**
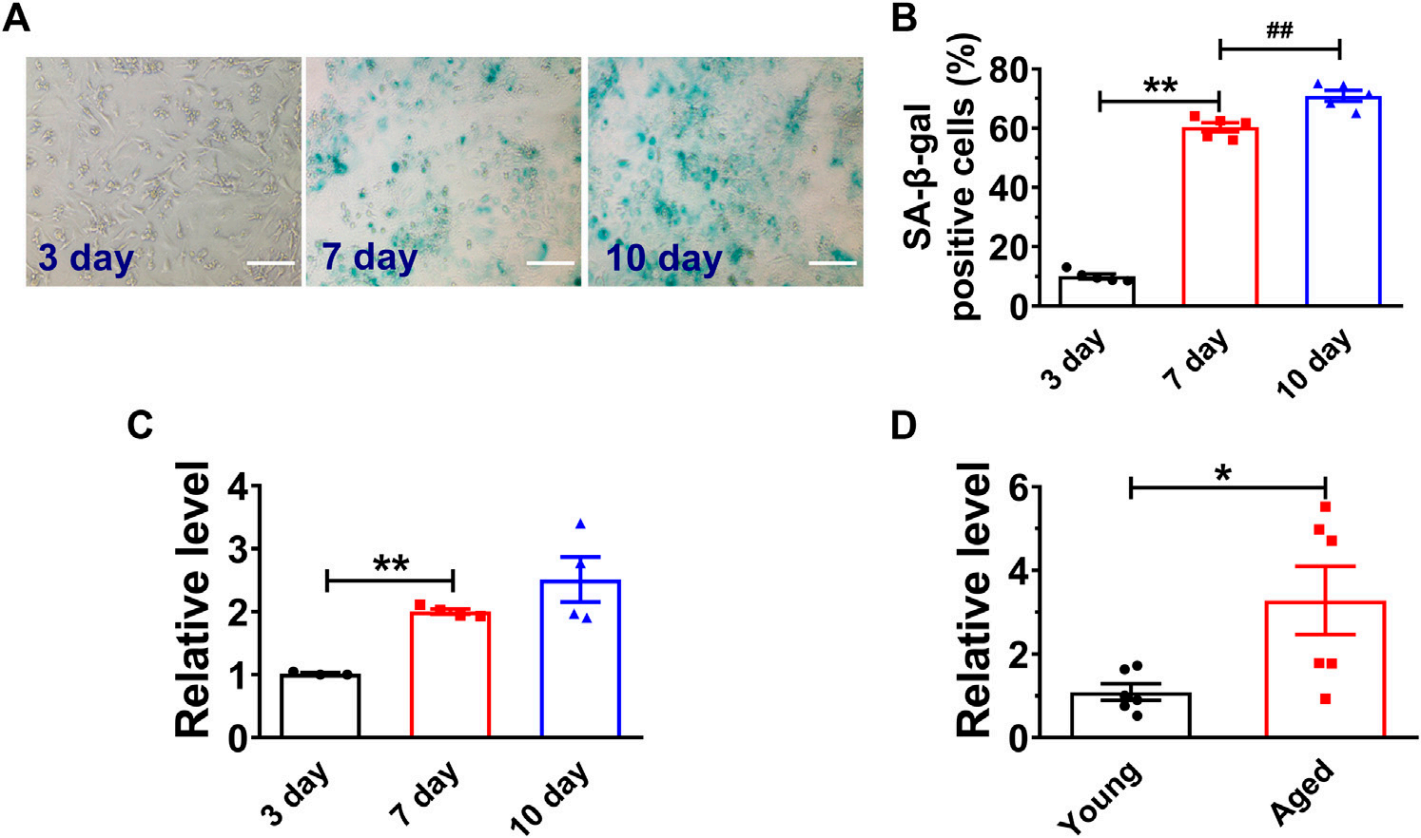
H19 is upregulated in senescent cardiomyocytes and aged mice heart. **(A,B)** Representative images of SA-β-gal staining and the averaged results showing the time-dependent increases in the percentage of SA-β-gal positive cells in NMVCs under high glucose conditions. Scale bars = 50 μm. **(C)** Time-dependent increases in the expression level of H19 in cardiomyocytes with varying exposure durations to high glucose. **(D)** Increased expression of H19 in aged mice hearts. Data are expressed as mean ± SEM, *p < 0.05 vs. Young, **p < 0.01 vs. 3 days, ^##^ *p* < 0.01 vs. 7 days, n = 5 or 6.

High glucose has been demonstrated to induce senescence in various cell types (E et al., 2014; Zhang et al., 2017a). We first analyzed the H19 expression in both high glucose (25 mM) treated NMVCs and aged mouse hearts (18 months old Kunming mice, half male and female) (Chang et al., 2015; Chen et al., 2019). As shown in Figures 1A,B, high glucose increased the number of senescence-associated β-galactosidase (SA-β-gal) positive NMVCs in a time dependent manner. SA-β-gal activity is the most widely used to identify senescent cells (Collado et al., 2007). In addition, the expression of H19 was also significantly increased in these senescent NMVCs (Figure 1C). Consistently, the expression of H19 was significantly increased in the hearts from 18 months old mice. (Figure 1D). These data suggested a potential functional role of H19 in the process of cardiomyocyte senescence.

### Overexpression of H19 Accelerates, Whereas Knockdown of H19 Expression Inhibits NMVC Senescence

**FIGURE 2:**
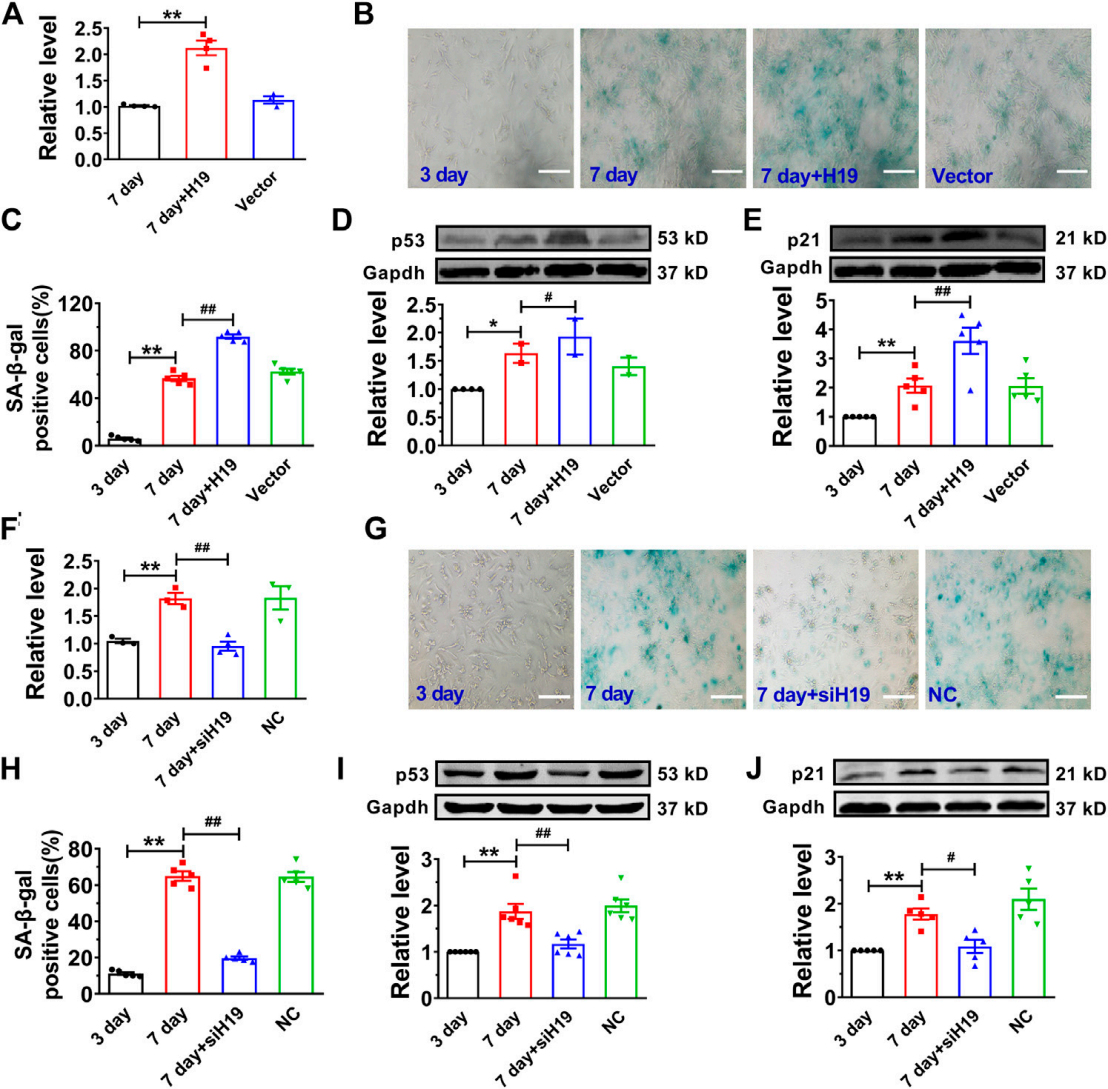
Effects of H19 on cardiomyocyte senescence. **(A)** Verification of H19 overexpression in cardiomyocytes transfected with H19-carrying lentivirus. **(B)** Representative images showing the effect of H19 overexpression on the SA-β-gal activity in senescent cardiomyocytes. Scale bars = 50 μm. **(C)** The averaged results showing increased SA-β-gal positive cells in H19 overexpression group. **(D,E)** After transfection with H19 lentivirus, the expression of age-associate protein p53 and p21 was increased in senescent cardiomyocytes. **(F)** Verification of the efficacy of H19 siRNA (siH19) in knocking down the expression of H19 in senescent cardiomyocytes. **(G)** Representative images showing the suppressive effect of siH19 on the SA-β-gal activity of senescent cardiomyocytes. Scale bars = 50 μm. **(H)** The averaged results showing decreased SA-β-gal positive cells in siH19 group. **(I,J)** After transfection with siH19, the expression of senescence-associated proteins p53 and p21 were decreased in senescent cardiomyocytes. Data are expressed as mean ± SEM, **p < 0.01 vs. 3 days, ^#^ *p* < 0.05 vs. 7 days, ^##^ *p* < 0.01 vs. 7 days, n = 4–6.

Then, we performed gain- and loss-of-function studies in NMVCs to investigate the effect of H19 on cardiomyocyte senescence. We introduced the lentivirus vector carrying the H19 gene into senescent cardiomyocytes for overexpression and obtained a 2.3-fold increase of H19 level (Figure 2A). We found forced expression of H19 markedly accelerated cardiomyocyte senescence as revealed by the increased number of SA-β-gal-positive cells after high glucose treatment for 7 days (Figures 2B,C). Correspondingly, senescence-associated proteins p53 and p21 were also significantly increased upon H19 overexpression, while no changes were observed in the control lentivirus group (Figures 2D,E). In contrast, H19 knockdown produced anti-senescence effects in cardiomyocytes. As illustrated in Figure 2F, H19 siRNAs (siH19) knocked down endogenous H19 and significantly inhibited the SA-β-gal positive senescent cardiomyocytes (Figures 2G,H). Consistently, siH19 also abrogated the increase in the expression of p53 and p21 in senescent cardiomyocytes (Figures 2I,J). These data indicated that H19 is capable of promoting the senescence response in cardiomyocytes.

### H19 Functions as a miR-19a ceRNA in Cardiomyocytes

**FIGURE 3:**
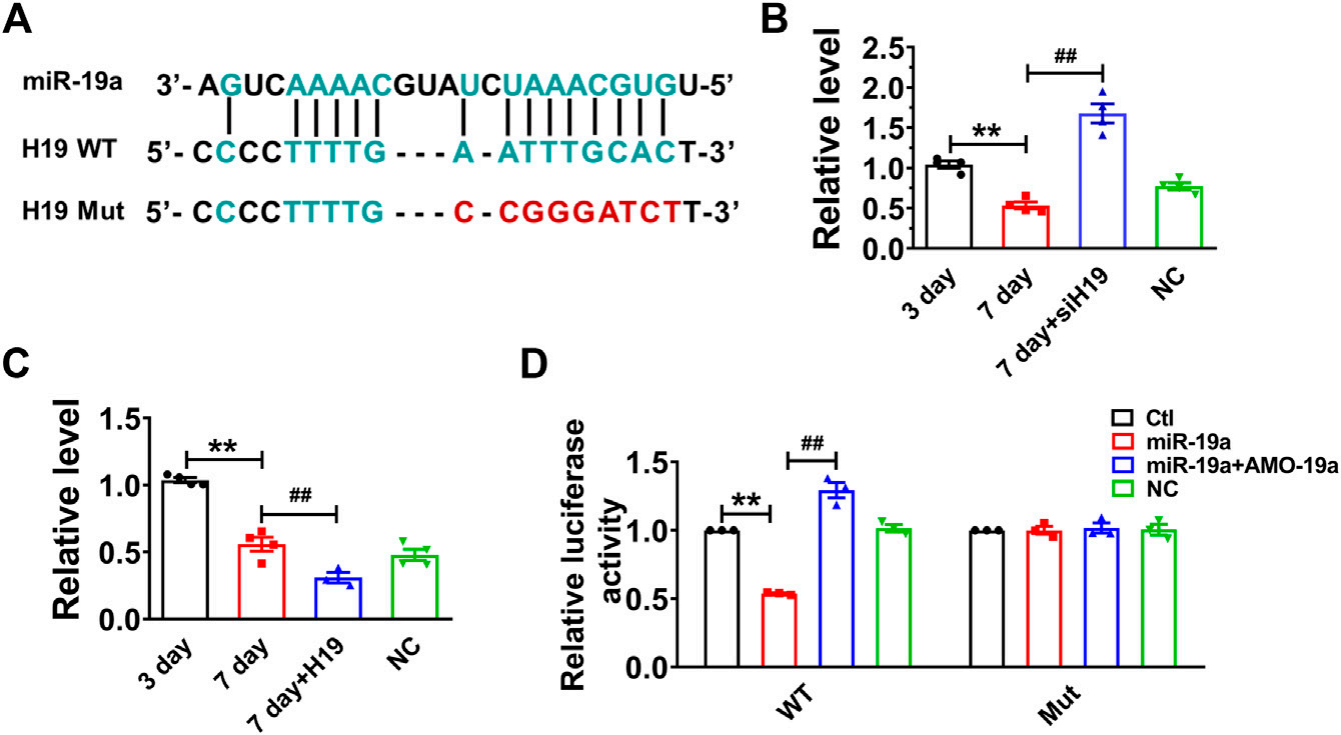
H19 acts as a ceRNA to miR-19a in cardiomyocytes. **(A)** Sequence alignment showing the base-pairing between H19 and miR-19a. **(B)** miR-19a was increased in siH19 group. **(C)** miR-19a was decreased after H19 overexpression in cardiomyocytes treated with high glucose for 7 days. **(D)** miR-19a strongly inhibited the luciferase activity of reporter plasmid containing H19 fragment compared with mutation group. Data are expressed as mean ± SEM, **p < 0.01 vs. 3 days/Ctl, ^##^ *p* < 0.01 vs. 7 days/miR-19a, n = 5.

Recent studies have reported that lncRNAs can act as ceRNAs to reduce the functional availability of miRNAs by the sequence complementarity mechanism, thereby reducing the target miRNAs (Cesana et al., 2011; Wang et al., 2014). It has been demonstrated that p53 functions as a downstream of miR-19 family members (miR-19a and miR-19b) in mediating MCF-7 cell proliferation (Li et al., 2014). Bioinformatics analysis (RegRNA 2.0) showed there are potential binding sites between H19 and miR-19 family members. Further studies showed that the expression of miR-19a was highly expressed and remarkably upregulated in senescent cardiomyocytes. Thus, we examined whether H19 acts as a ceRNA to sponge miR-19a in NMVCs. Our data showed that miR-19a acted as a possible target miRNA for H19 by silico analysis (Figure 3A). Indeed, knockdown H19 by its siRNA significantly increased miR-19a level, while overexpression of H19 significantly inhibited miR-19a level in NMVCs (Figures 3B,C). Luciferase reporter assay showed that co-transfection of the luciferase vector carrying the wild-type H19 fragment (Luc-wt-H19) and miR-19a mimic into HEK293 cells caused enormous diminishment relative to Luc-wt-H19 alone. The ability of miR-19a to suppress luciferase activities was dumbed when the complementary sequence in H19 was destroyed by nucleotides substitution mutation (Luc-mu-H19; Figure 3D). These data indicated that lncRNA H19 inhibits miR-19a expression by acting as its ceRNA.

### miR-19a Regulates Expression of p53 and p21 in Senescent Cardiomyocyte

**FIGURE 4:**
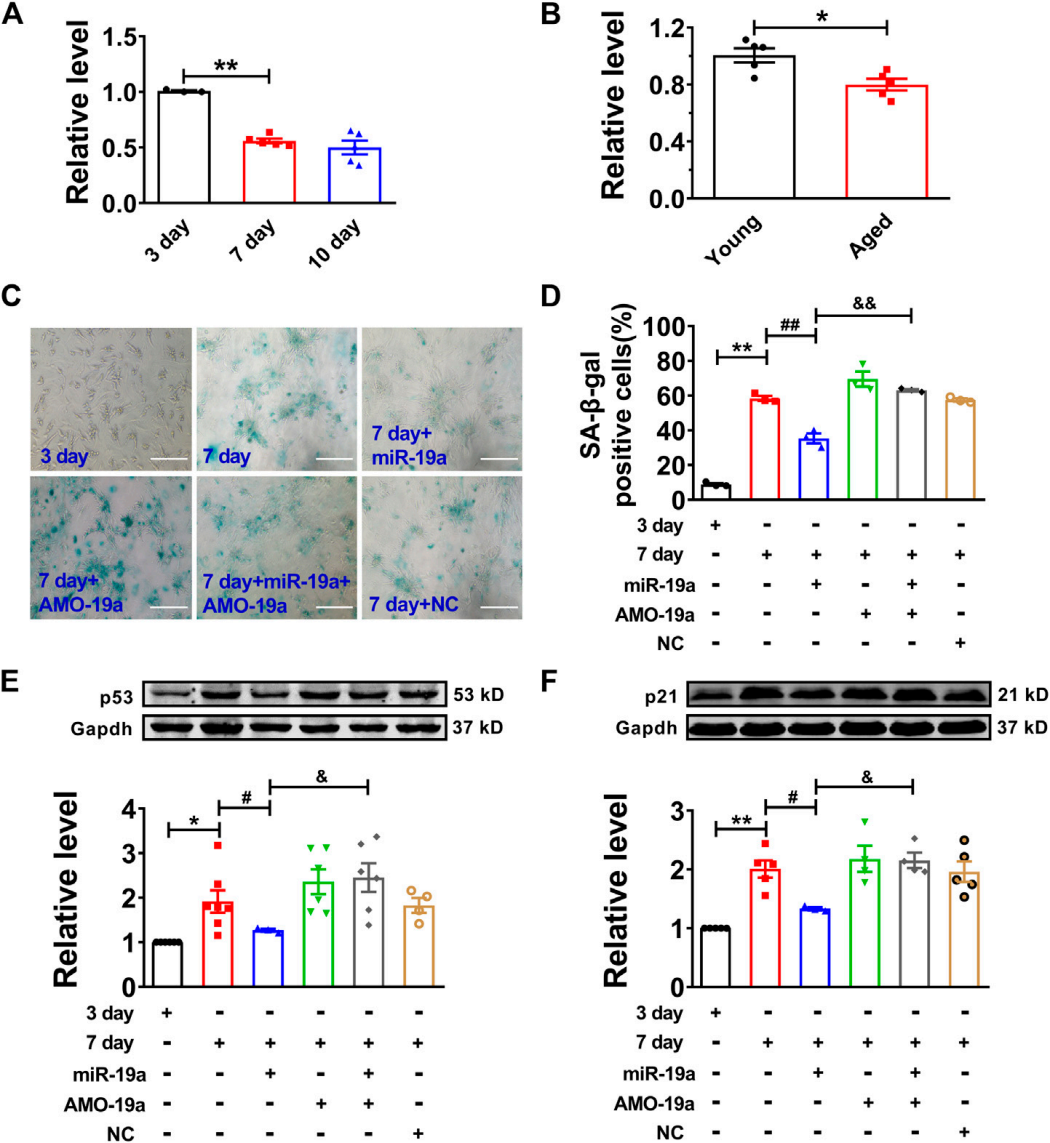
miR-19a involved in regulation of cardiomyocyte senescence. **(A)** Decreased expression of miR-19a with varying exposure durations to high glucose. **(B)** Decreased expression of miR-19a in aged mouse hearts. **(C)** Representative pictures showing the inhibitory effect of miR-19a on SA-β-gal activity of senescent cardiomyocytes. Scale bars = 50 μm. **(D)** The statistical results of SA-β-gal positive cells in different groups. **(E,F)** The suppressive effect of miR-19a on the expression of senescence-associated protein p53 and p21 in senescent cardiomyocytes. Data are expressed as mean ± SEM, *p < 0.05 vs. Young/3 days, **p < 0.01 vs. 3 days, ^#^ *p* < 0.05 vs. 7 days, ^##^ *p* < 0.01 vs. 7 days, ^&^ *p* < 0.05 vs. 7 days + miR-19a, ^&&^ *p* < 0.01 vs. 7 days + miR-19a, n = 3–6.

Then, we assessed the role of miR-19a in senescent cardiomyocytes. Our data showed that miR-19a expression was significantly decreased in both senescent cardiomyocytes and aged mouse hearts (Figures 4A,B). Moreover, overexpression of miR-19a significantly inhibited the number of SA-β-gal-positive cells and the p53 and p21 expression in senescent cardiomyocytes. In addition, AMO-miR-19a (AMO-19a) to knockdown endogenous miR-19a restored the high glucose-induced number of SA-β-gal-positive cells and the p53 and p21 expression (Figures 4C–F). Accordingly, these data indicated that miR-19a plays positive role in senescent NMVCs.

### SOCS1 Functions as Direct Target of miR-19a in Senescent Cardiomyocyte

**FIGURE 5:**
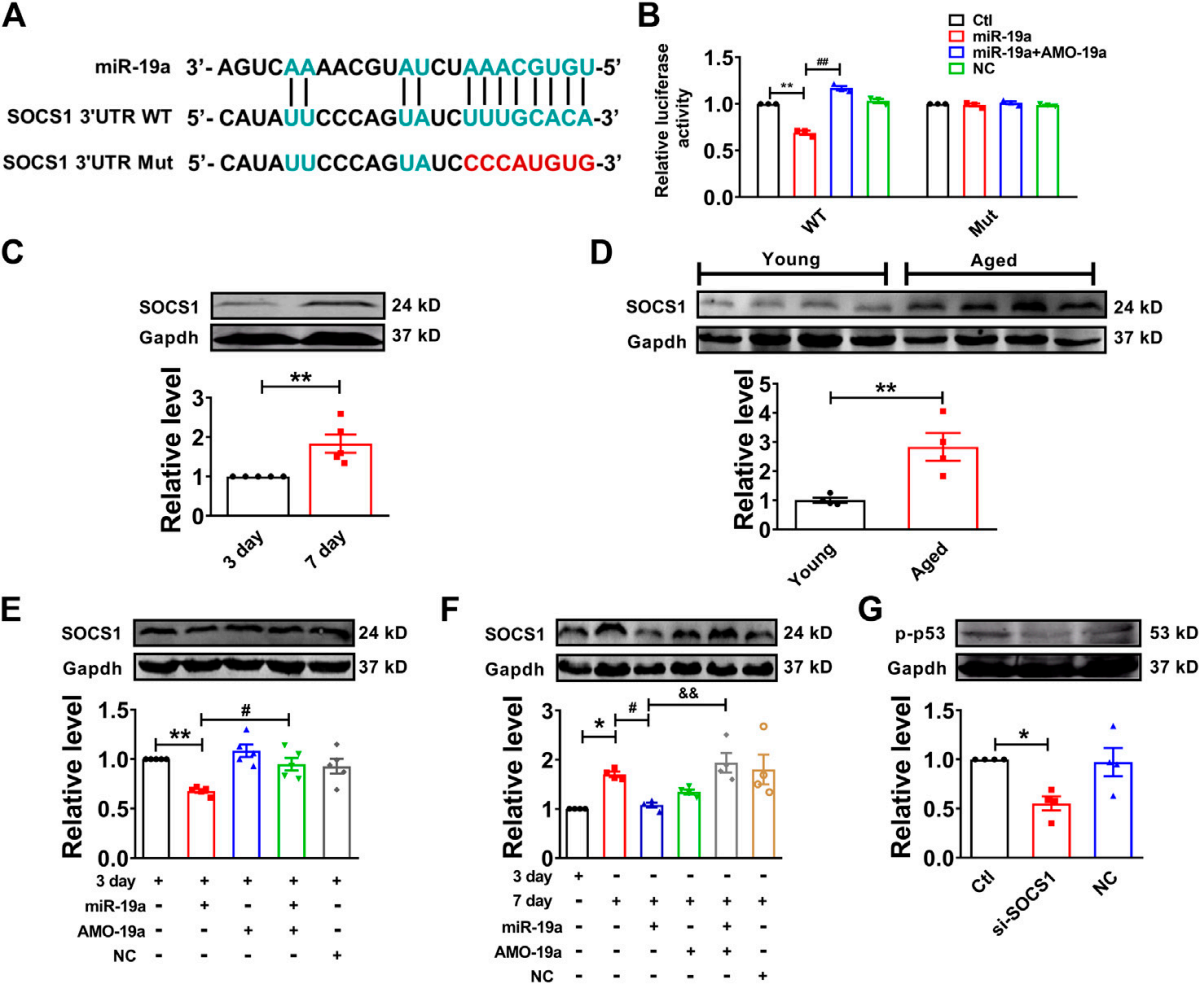
miR-19a targets to SOCS1 mRNA in cardiomyocytes. **(A)** Sequence alignment showing the base-pairing between miR-19a and SOCS1. The complementary base pairs in SOCS1 3′UTR and miR-19a are outlined in green; the mutated base pairs in SOCS1 3′UTR are outlined in red. **(B)** miR-19a strongly inhibited the luciferase activity of reporter plasmid containing SOCS1 mRNA fragment compared with mutation group. **(C)** SOCS1 was increased in senescent cardiomyocytes. **(D)** SOCS1 was enhanced in aged mouse hearts. **(E)** The suppressive effect of miR-19a on the expression of senescence-associated protein SOCS1 in cardiomyocytes at 3 days. **(F)** After transfection with miR-19a mimic, the expression of SOCS1 decreased cardiomyocytes at 7 days. **(G)** Effect of SOCS1 on the phosphorylation of p53 (p-p53). Data are expressed as mean ± SEM, *p < 0.05 vs. 3 days, **p < 0.01 vs. 3 days, ^#^ *p* < 0.05 vs. 7 days, ^##^ *p* < 0.01 vs. 3 days + miR-19a, ^&^ *p* < 0.05 vs. 7 days + miR-19a + AMO-19a, n = 5–6.

In order to investigate the target gene of miR-19a, we employed Targetscan to screen out the target factor of miR-19a and turned out to be SOCS1 as candidate (Figure 5A), which has been reported to induce cell senescence through p53 (Calabrese et al., 2009). Our luciferase reporter assay showed co-transfection of miR-19a with the luciferase reporter vector into HEK293 cells caused a dramatic decrease in luciferase activity compared with transfection of the luciferase NC vector alone. The miR-19a-induced inhibition of luciferase activity was rescued by AMO-miR-19a. However, miR-19a failed to affect the luciferase activity elicited by the construct carrying the SOCS1 3′-UTR with the mutant miR-19a site (Figure 5B). Moreover, the expression of SOCS1 was significantly increased in both senescent NMVCs and aged mouse hearts (Figures 5C,D). Consistently, miR-19a mimic significantly inhibited the expression of SOCS1, whereas AMO-miR-19a reversed the effect in 3-days or 7-days cultured cells (Figures 5E,F). In addition, SOCS1 siRNA significantly inhibited the phosphorylation of p53 (Figure 5G). These results, together with Figure 4, indicated that H19 competitively inhibited the binding of SOCS1 3′-UTR to miR-19a.

### H19 Regulates Cardiomyocytes Senescence via miR-19a/SOCS1/p53 axis

**FIGURE 6:**
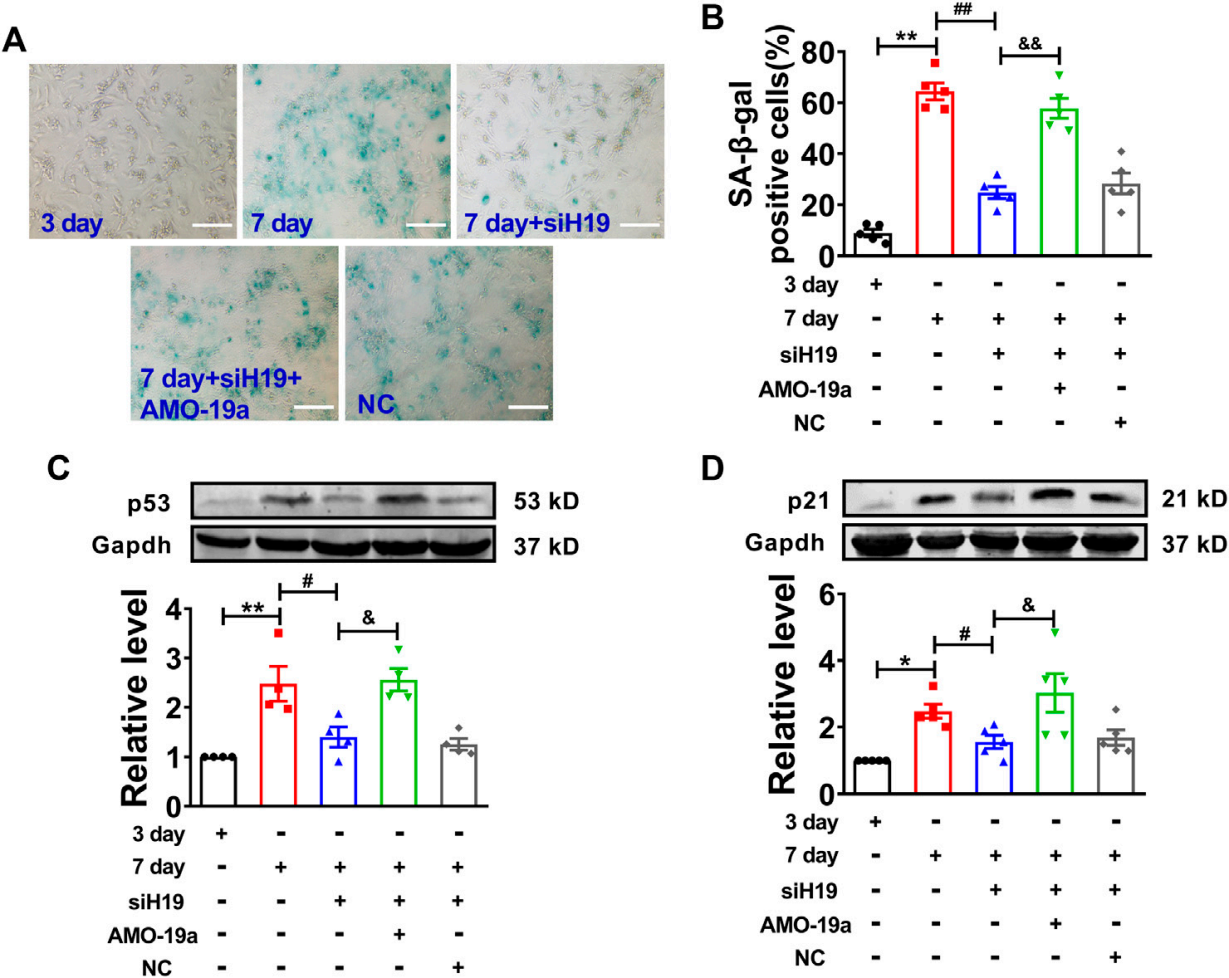
H19 accelerates cardiomyocyte senescence through inhibiting miR-19a. **(A)** AMO-19a counteracted the suppressive effect of siH19 on SA-β-gal positive cells. Scale bars = 50 μm. **(B)** The mean data showing that knockdown of miR-19a increased SA-β-gal positive cells inhibited by siH19 in senescent cardiomyocytes. **(C,D)** After transfection of AMO-19a, siH19 effect on the expression of senescence-associated protein p53 and p21 was counteracted in senescent cardiomyocytes. Data are expressed as mean ± SEM, *p < 0.05 vs. 3 days, **p < 0.01 vs. 3 days, ^#^ *p* < 0.05 vs. 7 days, ^##^ *p* < 0.01 vs. 7 days, ^&^ *p* < 0.05 vs. 7 days + siH19, ^&&^ *p* < 0.01 vs. 7 days + siH19, n = 5.

To further determine the molecular mechanism of H19 regulating cardiomyocytes senescence, knockdown of miR-19a by AMO-miR-19a mitigated the reduction of SA-β-gal-positive cells induced by H19 siRNA in senescent cardiomyocytes (Figures 6A,B). Furthermore, AMO-19a effectively prevented the downregulation of p53 and p21 induced by H19 siRNA in senescent cardiomyocytes compared with AMO-NC group (Figures 6C,D). These results indicated that H19 aggravates cardiomyocytes senescence via acting as a ceRNA for miR-19a to target the SOCS1/p53/p21 axis.

## Discussion

The present study, for the first time, shows that lncRNA H19 was significantly increased in senescent NMVCs and aged mouse hearts. Overexpression of H19 significantly promoted cardiomyocyte senescence, whereas knockdown of H19 inhibited cardiomyocyte senescence. Our data show that H19 acts as competing endogenous RNA (ceRNA) to regulate suppressor of cytokine signaling 1 (SOCS1) expression by sponging miR-19a subsequently leading to cardiac senescence by stimulation of the p53/p21 signaling pathway.

LncRNAs have been demonstrated to possess important functions in various biological processes, such as epigenetic modulation, transcriptional and post-transcriptional regulation (Feng et al., 2006; Wang et al., 2016). It has been reported that lncRNAs participate in the regulation of cell senescence (Azzalin et al., 2007; Abdelmohsen et al., 2013; Cao et al., 2020). MALAT1 and MIAT, known as the best-annotated lncRNAs, exhibit lower expression levels in senescent WI-38 cells. Silencing MALAT1 or MIAT in fibroblasts results in an increase in the number of senescent fibroblasts and protein markers of senescence p53 and p21 (Abdelmohsen et al., 2013). TERRA, a telomeric repeat-containing lncRNA, is involved in the regulation of the telomeric DNA damage response (Azzalin et al., 2007). However, the specific regulatory mechanism of lncRNA in cardiomyocyte senescence has not been fully understood.

The expression of H19 is enriched in the embryonic stage and responds to various stress conditions (Gabory et al., 2009; Monnier et al., 2013). H19 expression is deregulated in many tumors and other diseases (Wu et al., 2018; Fattahi et al., 2020). For example, in breast cancer cells, H19 directly regulates the target gene p53 to affect the proliferation of breast cancer cells (Yang et al., 2020). We have reported that H19 as an endogenous sponge for miR-877 mitigates myocardial I/R injury in mouse model (Li et al., 2019). It has been demonstrated that H19 regulates biological or pathological processes through different mechanisms such as interaction with proteins, as endogenous sponge for miRNAs and so on. In the present study, we found that H19 in senescent cardiomyocytes and aged hearts was significantly increased and suppression of H19 ameliorated the phenotypes of senescent cardiomyocytes; forced expression of H19 promoted NMVC senescence. Additionally, the expression of aging marker proteins p53 and p21 was decreased by transfection of H19 siRNA, indicating the contribution of H19 to cardiomyocyte senescence via regulating p53 and p21.

LncRNAs can act as ceRNAs to regulate expression of miRNAs, therefore influencing the activity and expression of miRNA-targeted protein-coding genes (Cesana et al., 2011; Wang et al., 2014). The present study experimentally established H19 functions as a ceRNA for miR-19a. SOCS1 is reported to be sufficient to induce p53-dependent senescence in fibroblasts through acting as adaptor for phosphorylation of p53 (Calabrese et al., 2009; Mallette et al., 2010). Luciferase assay showed that miR-19a interacted with 3′UTR of SOCS1 by complementary binding manner. miR-19a was significantly decreased in senescent cardiomyocytes. miR-19a reduced the number of senescent cardiomyocytes and decreased the protein levels of the senescence associated genes SOCS1 and its downstream factors p53 and p21. By comparison, silencing miR-19a had little effect on the senescence induced by high glucose. A possible explanation for this observation is that the senescence process had decreased the miR-19a down to the minimum level and further downregulating miR-19a by AMO-19a could hardly add any more effect on senescence. These data suggest that miR-19a plays a critical role in regulation of cardiomyocyte senescence and SOCS1, p53 and p21 are downstream factors or target genes of miR-19a.

Our study may represent the first to establish the relationship between H19 and miR-19a and their roles in cardiomyocyte senescence. The fact that miR-19a was able to reverse the senescence-promoting effects of H19 suggests that H19 acted via regulating the function of miR-19a thereby the downstream factor SOCS1. Moreover, it has been reported that aging is accompanied by enhancement of apoptosis in several organs, such as heart and brain (Tower, 2015). Studies have demonstrated that cardiomyocyte apoptosis is increased in aged mouse hearts (Gallogly et al., 2010; Hua et al., 2015). In our study, we found that overexpression of H19 augmented cardiomyocytes senescence and apoptosis, while silencing H19 showed inhibitory effects in senescence and apoptosis (data not shown). These data indicate that senescence and apoptosis have a very close relation likely due to senescence and apoptosis sharing common pathway to a certain degree. Undeniably, understanding the relationship between senescence and apoptosis has more implication for intervention of aging.

Our findings that either H19 siRNA or miR-19a mimic could abrogate the cardiomyocyte senescence further indicate that the senescence process might be attenuated or reversed, and H19 could be considered the potential molecular targets for relieving cardiomyocyte senescence and the associated cardiac disorders. However, more studies are needed to establish the case. In addition, it should be noted that the results of the present study are limited to the cellular level and future studies with *in vivo* animal models are absolutely required to verify our data.

In summary, this study shows that H19 was increased in senescent cardiomyocytes and aged mouse heart tissue and regulated the senescent phenotypes of cardiomyocytes through the miR-19a/SOCS1/p53/p21 pathway. Our study reveals a new mechanism of cardiomyocyte senescence and provides a new target for the treatment of cardiac senescence associated disease.

## Data Availability

The raw data supporting the conclusions of this article will be made available by the authors, without undue reservation.
